# The Role of Long Noncoding RNAs in Modulation of Stress Granules in Cancer

**DOI:** 10.1111/jcmm.71261

**Published:** 2026-06-29

**Authors:** Nazlı Şevval Menemenli, Bünyamin Akgül

**Affiliations:** ^1^ Noncoding RNA Laboratory, Department of Molecular Biology and Genetics Izmir Institute of Technology Izmir Türkiye

**Keywords:** cancer, chemotherapy, long noncoding RNAs, stress, stress granules

## Abstract

Stress granules are dynamic cellular structures that arise in response to stress. They play an important role in cancer cell survival by modulating multiple stress responses. Long noncoding RNAs (lncRNAs) have been identified as crucial regulators of stress granule (SG) dynamics, influencing cancer development and treatment resistance. LncRNAs play a role in the development and stability of stress granules, thereby enhancing cancer cells' ability to withstand severe conditions, such as chemotherapy. LncRNAs may promote the accumulation of pro‐apoptotic proteins within stress granules, thereby contributing to cancer cell persistence and potentially serving as a barrier to effective treatment. Recent findings highlight the significance of intricate interactions among lncRNAs, stress granules, and the tumour microenvironment (TME), underscoring the importance of targeting lncRNAs within stress granules to enhance the efficacy of current therapies. This review examines the role of lncRNAs in SG dynamics and their implications for cancer, with a focus on how lncRNAs regulate SG formation, function, and cancer cell resilience to stress.

## 1 Introduction

The ability of cancer cells to adapt to and resist various stimuli is crucial for their survival in changing environments [1, 2]. Cancer cells commonly respond to harsh conditions by rearranging cellular structures and reprogramming gene expression. In this endeavour, cellular compartmentalisation via membranes and proteins enables the execution of cellular responses within specific microenvironments. Recent studies have shown that cellular compartmentalisation can also be achieved through membrane‐less organelles [3].

The Integrated Stress Response (ISR) is an evolutionarily conserved adaptation mechanism activated by various cellular stressors, such as hypoxia, nutrient deficiency, and viral infection. Activation of stress‐sensitive kinases such as general control nonderepressible‐2 kinase (GCN2 kinase), PKR‐like ER kinase (PERK), heme‐regulated eukaryotic translation initiation factor 2a (eIF2a) kinase (HRI), and double‐stranded RNA‐dependent protein kinase (PKR) via dimerisation and autophosphorylation leads to phosphorylation of eIF2α at the Ser51 residue, integrating different stress signals into a common downstream translational control axis. Importantly, translational repression induced by eIF2α phosphorylation is a key trigger of stress granule (SG) formation. In this context, ISR directs not only the reprogramming of gene expression but also the dynamic organisation of ribonucleoprotein structures where mRNA is temporarily stored and its fate determined [4, 5, 6].

SGs are membrane‐free ribonucleoprotein (RNP) aggregates found in the cytoplasm and formed in response to a range of stress stimuli, including oxidative damage, hypoxia, and nutritional restrictions [7, 8]. SGs play a crucial role in translation regulation, RNA metabolism, and cell survival, particularly in cancer. To this end, SGs are essential for maintaining non‐essential mRNAs, regulatory proteins, and non‐coding RNAs (ncRNAs), including long non‐coding RNAs (lncRNAs). Among many RNA species that exist in SGs, lncRNAs have emerged as important regulators. LncRNAs, which lack the protein‐coding potential, have been linked to cancer formation, metastasis, and drug resistance [9]. Understanding the interplay between lncRNAs and cancer‐associated SGs is crucial for developing new therapeutic options, given the rising evidence of their involvement [10]. In this review, we aim to highlight the heterogeneity of the lncRNA components of SGs and their potential role in cancer progression.

## 2 Heterogeneity of Stress Granules in Cancer Cells

SGs are cytoplasmic, complex RNP structures that develop during cellular stress to block the translation of mRNAs implicated in stress responses. They contribute significantly to cellular homeostasis and the stress response by preferentially encapsulating mRNAs and thereby supporting cell survival under harsh conditions. The phosphorylation of eIF2α by PERK/PEK, GCN2, PKR, and HRI is the primary mechanism for the canonical assembly of SGs [11]. These kinases can phosphorylate eIF2α, reducing the concentration of the eIF2‐GTP‐tRNA^Met^ ternary complex. When the ternary complex is decreased, the RNA‐binding proteins T‐cell intracellular antigen‐1 (TIA‐1) and T‐cell restricted intracellular antigen‐related protein (TIAR) promote the formation of the atypical first 48S complex. This complex cannot recruit the 60S ribosomal subunit for translation, but it can recruit SGs for assembly [12].

SGs are categorised as canonical and non‐canonical based on their lncRNA signatures and protein components, reflecting specialised functions in different cells and under stress conditions [13, 14]. Canonical SGs are constituted from the suppression of stress‐dependent translation of eIF2α activation. The mRNP that cannot initiate translation creates liquid–liquid phase dissociation (LLPS) as a result of the accumulation of the 48S pre‐initiation complex unit (PIC). Canonical SGs contain translation‐sequestered mRNAs, initiation factors such as eIF3, eIF4E, and eIF4G, 40S ribosomal subunits, and transcripts with long open reading frames (ORFs) and low translational activity. Proteins involved in the biogenesis of stress granules include the core nucleating proteins G3BP2 and G3BP1, PABP, cap‐binding proteins, TIA‐1, and TIAR [15]. Non‐canonical SGs generally form independently of eIF2α phosphorylation in the presence of eIF4A inhibitors, translation elongation blockade, or mTOR inhibition. Furthermore, in some cases they can form without ribosome disassembly or rely on 48S accumulation. They may be more stable, less dynamic and are often devoid of certain proteins, such as Importin α1, HDAC6 and RACK1. Similar to the canonical ones, they contain main nucleators such as G3BP1/2, but are devoid of the core canonical components. Non‐canonical SGs, unlike canonical structures, possess unique RNA recruitment dynamics; this difference resides primarily in the poly(A) mRNA content and the arrangement of the 5′‐cap epitranscriptome [16, 17].

The lncRNA‐associated SG subtypes contribute to the diversity of SG roles in normal physiology and disease. However, the recruitment of specific mRNA components and protein cofactors remains mainly unknown in stress‐specific canonical and/or non‐canonical SG subtypes [13, 14]. As characterised SG subtypes, UV SGs are RNA‐depleted and protein‐driven, with canonical SG markers, but differ from classical SGs induced by arsenite or heat shock. These non‐canonical granules lack eIF3, and their biogenesis is independent of eIF2α phosphorylation [18].

SGs help cells survive under harsh conditions, such as hypoxia and chemotherapy, which can promote cancer development and therapeutic resistance [19]. SGs improve cell growth, resistance to metabolic stress, and migration under anoikis, a condition in which cells lose adhesion to the extracellular matrix [20]. SUCLA2‐associated SGs facilitate the translation of antioxidant enzymes, such as catalase, thereby reducing oxidative stress within cancer cells and enabling them to avoid anoikis [21]. Several oncogenic pathways, including KRAS, PI3K, TORC1, and HDAC6, have been linked to increased SG assembly. KRAS‐mutated colorectal cancer cells have a greater tendency to form SGs than wild‐type cells, possibly due to COX2 overexpression via the MAPK pathway [22]. While SGs have been linked to various oncogenic processes, their widespread occurrence and impact in tumours with KRAS mutations remain unknown. Actually, a recent study reports that SGs are absent and do not have an impact on cancer progression in tumours with KRAS mutations, at least not universally [23]. However, there is a paradigm shift in SG‐mediated modulation of cancer in that SGs are not merely cytoprotective organelles. For example, the Y‐Box Binding Protein 1 (YBX‐1)‐Ras‐GTPase‐activating protein SH3 domain‐binding protein 1 (G3BP1) axis of the stress response machinery affects cancer growth, providing numerous survival benefits, such as apoptosis resistance, increased proliferation, EMT induction, chemotherapy resistance, and immune evasion [24, 25, 26].

As an essential regulator for SG assembly, Ras‐GTPase‐activating protein SH3 domain‐binding protein 2 (G3BP2) has been related to tumour formation in breast cancer [27], where it modulates the expression of stem‐cell‐associated genes such as Oct‐4 and Nanog via its interaction with SART3, promoting tumour growth and proliferation [28]. Besides G3BP2, nucleating proteins like G3BP1 and cell cycle‐associated protein 1 (CAPRIN1), which act as important regulators of SG dynamics, manage SG assembly [29]. Notably, reduced G3BP1 expression in U87 cells drastically decreases SG formation while enhancing the apoptotic response to bortezomib, shown by increased Caspase‐3 activity. Targeting these nucleating proteins could be a useful strategy for combating chemotherapeutic resistance [29, 30].

Although many studies indicate that SGs are cytoprotective condensates that facilitate cellular adaptability to harsh environments, SG production may not always benefit cell survival. Granule composition, cellular environment, stress level, and duration all seem to have an impact on the functional outcomes of SG assembly. Consequently, SGs may have either pro‐survival or pro‐apoptotic effects depending on the particular molecular environment in which they are formed [31, 32]. To this extent, ultraviolet (UV)‐induced SGs have unique compositional and signalling traits in contrast to classical SGs induced by oxidative stress or nutrient depletion. In contrast to traditional eIF2α‐dependent pathways, these granules are often linked to apoptosis and irreversible cellular damage rather than recovery and survival. These findings imply that some condensate compositions may actively support cell death programmes [33, 34].

There is still much to learn about how lncRNAs influence these variable SG‐mediated outcomes. However, a number of lncRNAs that control stress signalling, senescence, and apoptosis may be modulators of non‐canonical SG responses. For example, in several cancer models, it has been demonstrated that the tumour suppressor lncRNA *MEG3* activates p53 signalling, increases the transcription of pro‐apoptotic genes, and inhibits tumour growth. *MEG3*'s capacity to alter stress‐responsive signalling pathways suggests that tumour‐suppressive lncRNAs may modulate the SG composition toward pro‐apoptotic states rather than cytoprotective condensates [35, 36]. Similarly, by controlling p53‐dependent gene expression programmes, the lncRNA *lincRNA‐p21*, a transcriptional target of p53, licences apoptosis and prevents cellular growth. Another potential molecule that might indirectly affect the development or operation of SGs during genotoxic stress is *lincRNA‐p21*, given the close relationship between translational control, stress signalling, and SG assembly. Future research examining whether p53‐regulated lncRNAs are specifically drawn into SGs or alter SG‐associated RNA–protein networks may shed light on how SGs influence cell destiny [37].

**FIGURE 1:**
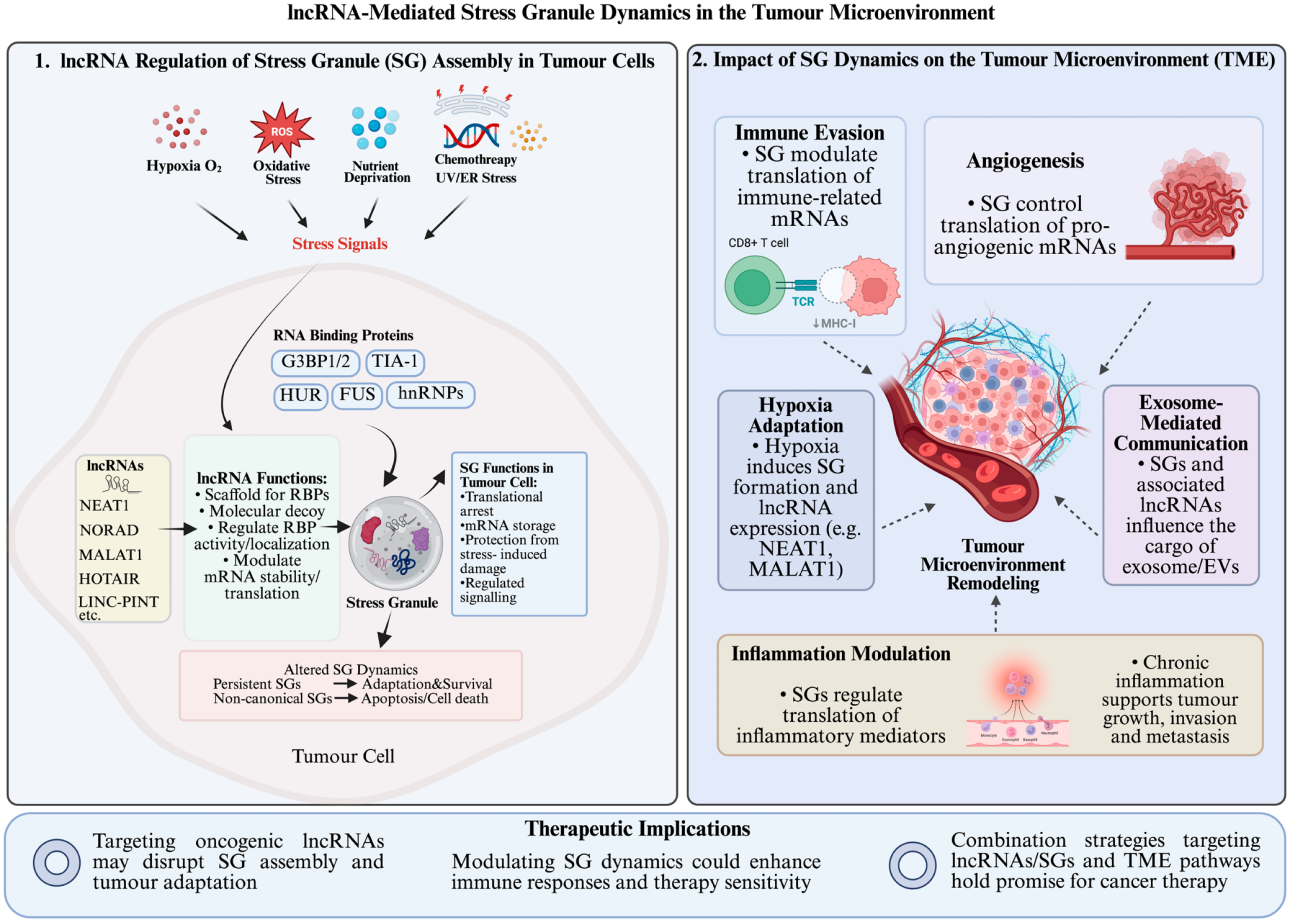
LncRNA‐mediated regulation of stress granules and their impact on the tumour microenvironment. Stress factors that encourage the development of stress granules (SG) in cancer cells include hypoxia, oxidative stress, nutritional deprivation, and stress brought on by therapy. Stress‐responsive lncRNAs, such as NEAT1, NORAD, MALAT1, HOTAIR, and LINC‐PINT, interact with RNA‐binding proteins (RBPs), modify mRNA translation, and modulate stress‐adaptive signalling pathways to affect SG assembly and function. By encouraging translational arrest, mRNA storage, and defence against stress‐induced damage, SGs aid in the survival of cancer cells. SG dynamics affect a variety of elements of the tumour microenvironment (TME) outside of cancer cells, such as immune evasion, angiogenesis, hypoxia adaptation, inflammation, and exosome‐mediated communication. Together, these mechanisms promote the growth of tumours and their resistance to treatment. Therefore, targeting the lncRNA–SG axis may be a promising therapeutic approach to improve treatment responses and overcome cancer cell adaptability [19, 38, 39, 40]. Created in https://BioRender.com.

A number of SG‐related lncRNAs, such as *NEAT1*, *NORAD*, and *MALAT1*, are typically associated with tumour survival, therapeutic resistance, genomic stability, and stress adaptation (Figure 1). The potential that SG‐associated lncRNA repertoires may be functionally biased toward cytoprotection is raised by the apparent prevalence of pro‐survival lncRNAs. It is unclear, though, if this observation represents a true biological preference or just the current focus of cancer‐focused research. To ascertain whether different classes of lncRNAs contribute to the establishment of functionally diverse SG populations, SG‐associated transcriptomes should be characterised under various cellular states, such as apoptosis, senescence, or irreversible growth arrest [41, 42, 43]. All of these findings point to a scenario where lncRNAs may contribute to the formation of non‐canonical condensates that support growth arrest or cell death in addition to facilitating adaptive SG responses. Thus, comprehending how lncRNAs influence SG composition under various stress scenarios may uncover hitherto unknown pathways connecting RNA condensates to decisions about cancer cells' fate.

Although SGs are typically considered intracellular stress‐adaptive structures, the emerging findings indicate that they may have a major impact on the tumour microenvironment (TME) in addition to serving as cell‐autonomous survival mechanisms (Figure 1). Strong inducers of SG assembly, such as hypoxia, food deprivation, oxidative stress, endoplasmic reticulum stress, and immune‐mediated stresses, are constantly present in tumour cells. As a result, SGs have evolved as a crucial adaptation mechanism that helps cancer cells endure the harsh environment within the TME. For example, SGs control immune evasion, angiogenesis, metabolic adaptation, therapeutic resistance, and tumour cell survival [19, 38]. Immune modulation is one of the most noteworthy connections between SGs and the TME. The inflammatory environment surrounding tumour cells can be rearranged by SGs' ability to selectively sequester signalling molecules, mRNAs, and RNA‐binding proteins involved in innate immune responses and cytokine production. Specifically, SG assembly has been linked to immune surveillance processes, stress‐induced inflammatory pathways, and interferon signalling modulation. By reducing the expression or translation of immune‐stimulatory genes while maintaining pro‐survival signalling networks, SGs may aid in immune evasion. It is possible that lncRNA‐mediated remodelling of SG dynamics indirectly contributes to the creation of an immunosuppressive milieu that promotes tumour progression [38, 44].

Another element of the TME that is intimately related to the SG biology is hypoxia. In addition to supporting translational reprogramming that improves cellular tolerance to oxygen deprivation, hypoxic stress serves as a powerful inducer of SG formation. SGs can boost angiogenic responses and metabolic flexibility by selectively regulating genes involved in hypoxia‐responsive pathways, including those controlled by HIF signalling. In this regard, lncRNAs, such as *MALAT1* and *NEAT1* have been linked to angiogenesis, tumour growth, and hypoxia adaptation. These lncRNAs' established roles in stress adaptation imply that they may affect the recruitment, stabilisation, or translational control of pro‐angiogenic transcripts within SG‐associated regulatory networks, despite the lack of direct evidence connecting them to SG‐dependent regulation of angiogenic signalling. For elements like VEGFA and other hypoxia‐responsive mediators that promote neovascularisation in solid tumours, this notion is especially pertinent [44, 45]. SGs may affect intercellular communication within the TME in addition to immune modulation and angiogenesis. A growing body of research indicates that SG‐associated RNA and protein networks may influence the makeup of extracellular vesicles and exosomal cargo, which may change how tumour cells, stromal cells, endothelial cells, and immunological populations communicate. SG‐associated lncRNAs may indirectly influence extracellular communication pathways that promote metastatic dissemination, stromal remodelling, and therapeutic resistance because lncRNAs often serve as molecular scaffolds for RNA‐binding proteins and take part in stress‐responsive ribonucleoprotein complexes. The growing convergence of extracellular signalling, biomolecular condensates, and lncRNA biology is a potential field for further research, despite the lack of direct mechanistic evidence [44, 46].

All of these findings point to the lncRNA–SG axis being more than just a regulator of intracellular stress adaptation. Instead, lncRNA‐mediated SG dynamics may contribute to the larger ecological environment of the TME by affecting immunological responses, angiogenic programmes, and intercellular communication networks. It is crucial to combine cutting‐edge technologies, such as SG transcriptomics, spatial profiling technologies, and TME‐focused functional analyses, to ascertain how particular lncRNAs change the composition of stress granules and whether these changes support immune escape, angiogenesis, or resistance to cancer treatments [19, 38].

The variability in SG biogenesis and function suggests that therapeutic efforts targeting SGs must be carefully tailored to the diverse cancer environments [47]. The content of SGs varies across cancer types, reflecting differences in oncogenic signalling, RNA‐binding protein levels, and lncRNA‐based regulatory networks. In KRAS‐focused pancreatic tumours, increased translational stress and elevated G3BP1 expression may promote the formation of more stable SGs, enhancing the sequestration of pro‐apoptotic transcripts and supporting chemoresistance. In contrast, in ovarian cancer cells, particularly A2780 and SKOV3, with high DEAD (Asp‐Glu‐Ala‐Asp)‐box helicase 3 X‐linked (DDX3X) levels, SGs are proposed to reprogramme the mitochondrial unfolded protein response, modulating the accessibility of adaptive factors such as ATF5 and influencing the response to PI3K/mTOR inhibitors. In variants where SG formation is impaired, apoptotic signals are not adequately buffered, potentially increasing therapeutic sensitivity [40, 48, 49]. These findings reveal that SG variability is not only quantitative but also compositional and functional, and that it shapes treatment outcomes depending on tumour type and context [48].

Cancer cells exploit SGs to withstand stress, reorganise their translational landscape, and avoid apoptosis. Therefore, modulating the development of SGs or targeting the assembly of functional SGs with specific molecules could be a possible therapeutic approach, either alone or in combination with other treatments (e.g., chemotherapy, radiotherapy) to restore the chemosensitivity of cancer cells [39, 50, 51]. SGs have been shown to have a protective role and are being investigated as a possible anticancer target in vitro and in vivo. Determining whether combining SG suppressors with anticancer medicines can help prevent drug resistance is therefore crucial [52].

## 3 Long Non‐Coding RNAs as Scaffolds in SG Formation

### 3.1 Long Non‐Coding RNAs as Stress Granule Modulators

LncRNAs are defined as RNA polymerase II‐transcribed molecules longer than 200 nucleotides, which are not translated into functional proteins. They are capped, spliced, and frequently polyadenylated, but lack conserved open reading frames. This wide term comprises an immense and diverse set of transcripts in terms of biogenesis and genetic origin including intergenic (lincRNAs), intronic, antisense, sense‐overlapping, and bi‐directional transcripts, along with promoter‐ and enhancer‐associated species (eRNAs), resulting in heterogeneous isoforms with lower abundance, higher tissue specificity, and enriched nuclear localisation when compared to mRNAs [53]. They originate from a variety of genomic locations. Their production and fate are influenced by promoter architecture, chromatin status, alternative splicing/3′‐end processing, and RNA decay pathways, each of which affects localisation and activity [54].

In the nucleus, lncRNAs act as signals, decoys, guides, or scaffolds to recruit or sequester transcription factors and chromatin modifiers, organise higher‐order chromatin, and regulate enhancer‐promoter communication; in the cytoplasm, they modulate mRNA stability and translation, serve as interaction hubs for RNA‐binding proteins, and participate in the formation and regulation of biomolecules [55, 56]. Collectively, all of these features position lncRNAs as diverse regulators at the transcriptional and post‐transcriptional levels, with increasing evidence of their participation in stress response pathways. Among their developing activities, lncRNAs play a particularly intriguing role in SG biogenesis, orchestrating RNA‐protein interactions that control condensate assembly and dynamics during physiological stress [57, 58, 59].

Since SGs are cytoplasmic ribonucleoprotein condensates, lncRNAs' subcellular location is probably a key factor in dictating their role in SG biology. Through physical interactions with cytoplasmically‐sequestered mRNAs and SG‐associated RNA‐binding proteins (RBPs), cytoplasmic lncRNAs are in a good position to actively contribute to the formation of SGs. Actually, the direct incorporation of lncRNAs into SGs may require cytoplasmic localisation, where they might operate as molecular scaffolding, modify RNA–protein interaction networks, or affect condensate composition and dynamics. Depending on the cellular context and stress conditions, cytoplasmic lncRNAs can either promote or inhibit the production of SGs through interactions with important SG nucleators, such as G3BP1, TIA‐1, TIAR, and FUS [53, 60].

It is not clear if lncRNAs will directly incorporate into cytoplasmic SGs under physiological conditions due to their mainly nuclear localisation. However, via controlling transcriptional and post‐transcriptional processes, nuclear lncRNAs may still have important, yet indirect, influence on SG biology. Nuclear lncRNAs, for instance, have the ability to modify chromatin accessibility, transcription factor activity, alternative splicing, RNA processing, and nuclear export, all of which have an impact on the abundance and biogenesis of transcripts that eventually make their way into the cytoplasmic SG pool. Furthermore, nuclear lncRNAs may indirectly influence SG biogenesis, persistence, and disassembly by controlling the expression of SG‐associated RBPs or signalling pathways implicated in cellular stress responses [61, 62].

Notably, a number of lncRNAs linked to stress responses, such as *MALAT1* and *NEAT1*, are primarily nuclear in basal conditions but have been shown to impact pathways related to cellular stress adaptation, RNA metabolism, and SG formation. These findings imply that a lncRNA does not always need to be directly localised within SGs in order to control the SG biology. Instead, lncRNAs may modulate the SG biology through a variety of regulatory mechanisms, from upstream control of gene expression programmes that influence the cellular stress response to direct involvement in cytoplasmic condensates [63, 64]. Cutting‐edge technologies, such as subcellular transcriptomics, stress granule purification techniques, and live‐cell imaging, may be merged to identify lncRNAs that physically localise within SGs and those that mainly control SG dynamics by indirect nuclear processes. These studies could be useful in uncovering if cellular localisation has a significant role in determining the functions of lncRNA in SG‐associated regulatory networks.

LncRNAs affect SG assembly and dynamics via interacting with SG proteins, such as G3BP1, G3BP2, FUS RNA‐binding protein (FUS), TIA1, TAR DNA‐binding protein (TDP‐43) and DDX3X, and other RNA molecules [59, 65]. They act as scaffolds, regulators, or SG components, influencing SG‐associated cellular responses to various stressors, including heat shock, hypoxia, DNA damage, and nutritional restriction [1, 2, 39]. Certain lncRNAs act as scaffolding molecules, enabling the recruitment of RBPs required for the assembly of SGs as they rapidly nucleate phase separation [66]. LncRNAs partition preferentially into stress granules, implying that their length may improve RNA–RNA interactions that aid assembly. In addition, their enrichment is associated with having the capacity to base‐pair with longer mRNAs, which contributes to the SG transcriptome [67].

LncRNA nuclear paraspeckle assembly transcript 1 (NEAT1) is essential for the development of paraspeckles, which are nuclear RNA‐protein complexes that function similarly to SGs. NEAT1 can also influence SG dynamics and the formation of paraspeckles during stress by assisting in the sequestration of mRNAs and RBPs such as SFPQ, NONO, PSPC1, and associations with TDP‐43 and FUS. The main regulatory mRNA components of paraspeckle function are inflammatory genes such as IL‐8, DNA damage and stress response genes, cell cycle regulators, and nuclear retention of A‐to‐I edited mRNAs [68, 69, 70]. Small nucleolar RNA host gene 8 (SNHG8), a long non‐coding RNA, helps generate stress granules by associating with the RNA‐binding protein TIA1. Through its direct interaction with TIA1, SNHG8 may function as an RNA scaffold that modulates phase separation dynamics and promotes stress granule nucleation. This mechanism provides transient cellular protection by intensifying translational repression during acute stress. However, granule stability maintained by SNHG8 can lead to the suppression of pro‐apoptotic translational circuits, facilitating stress adaptation and the development of therapeutic resistance in chronic or pathological conditions [65]. Dysregulation of SNHG8, particularly in tauopathies, leads to increased SG formation, suggesting a role in the pathogenesis of these neurodegenerative diseases such as Alzheimer's disease. While transient SGs support cellular adaptation under physiological conditions, in the context of tauopathies, over‐stabilised and dynamised granules can lead to chronic translational suppression, pathological protein aggregation, and increased neuronal fragility, playing a decisive role in Alzheimer's disease progression [65].

SGs are mostly composed of RBPs, which regulate RNA metabolism and play a major role in granule assembly. An essential regulatory layer controlling the formation, composition, and dynamics of SGs is the interaction between lncRNAs and RBPs. Certain RNA sequence motifs and structural components, such as certain stem loops, hairpins, bulges, and other higher‐order secondary structures, frequently mediate these interactions. Many lncRNAs, in contrast to mRNAs, have highly structured domains that allow for the selective recruitment of several RBPs at once, acting as molecular scaffolds that facilitate the assembly of RNP complexes. SG nucleation and maturation are facilitated by lncRNAs' ability to modify the local concentration of SG‐associated proteins and RNAs through their scaffolding roles [53, 71].

The existence of intrinsically disordered regions (IDRs) and low‐complexity domains (LCDs), which lack stable tertiary structures yet permit dynamic and reversible intermolecular interactions, is a characteristic shared by many SG‐associated RBPs. Extensive IDRs found in core SG nucleators, such as G3BP1, FUS, TDP‐43, TIA‐1, TIAR, and hnRNP family members, provide weak but multivalent interactions with RNA molecules and protein partners. The physicochemical foundation required for SG condensation is provided by these multivalent interactions, which also improves molecular communication throughout the cytoplasm. Crucially, lncRNAs can aid in this process by harbouring additional binding sites for RBPs, which raises interaction valency and promotes the development of higher‐order RNP networks. As a result, lncRNAs have been recently acknowledged as active modulators of SG architecture and stability in addition to being passive SG cargo molecules [32, 63, 72]. Liquid–liquid phase separation (LLPS), a biophysical mechanism that underlies the creation of many membraneless organelles, including SGs, is intimately associated with the cooperative interactions between lncRNAs and RBPs. LLPS comes about when biomolecules demix into concentrated condensates and dilute surrounding phases due to multivalent protein–protein, protein–RNA, and RNA–RNA interactions. Depending on their quantity, length, sequence constituents, and structural arrangement, RNA molecules can have both supportive and inhibitory effects on phase separation. In this regard, lncRNAs may function as molecular scaffolds that concentrate RBPs linked to SGs, control the viscosity and material characteristics of condensate, and affect the recruitment or exclusion of particular client proteins [64, 73, 74].

#### 3.1.1 lncRNA‐Encoded Micropeptides in Stress Adaptation and Cell Fate

LncRNAs have long been thought of as non‐protein‐coding transcripts that perform their activities primarily through structural or regulatory RNA‐protein and RNA–RNA interactions [53]. Recent data suggest that a considerable portion of lncRNAs includes cryptic short open reading frames (sORFs, < 300 nucleotides) with functional coding potential, leading to a paradigm change in this concept. Under certain environmental and stress conditions, these highly conserved sORFs elude normal translational surveillance and produce bioactive micropeptides, also known as sORF‐encoded peptides (SEPs) [75]. This dual functionality adds a new level of evolutionary and functional complexity to the lncRNA architecture, establishing these molecules as dual‐action players, which operate as structural scaffolding and translational templates during cellular crises [2].

**FIGURE 2:**
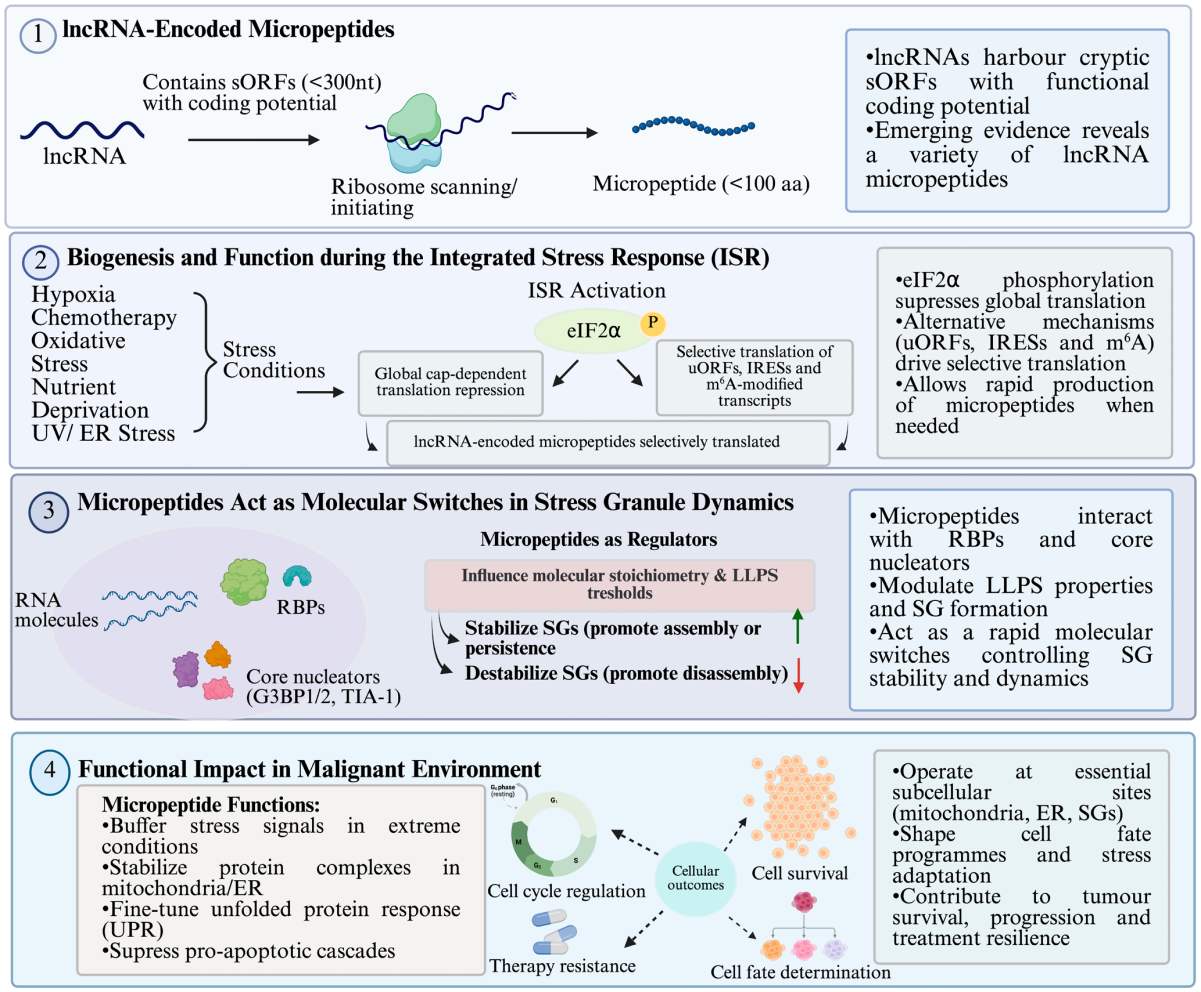
LncRNA‐encoded micropeptides may play a part in stress granule dynamics and stress adaptation. Functional micropeptides can be translated from long non‐coding RNAs (lncRNAs) that contain short open reading frames (sORFs). Activation of the integrated stress response (ISR) under cellular stress circumstances promotes the selective translation of particular transcripts, including lncRNA‐derived micropeptides, while suppressing global protein synthesis. By altering RNA‐binding proteins, nucleation mechanisms, and liquid–liquid phase separation (LLPS) characteristics, these micropeptides control the construction and disassembly of stress granules (SG). Micropeptides assist cancer progression by promoting cell survival, adaptability, and therapy resistance in malignant microenvironments through their effects on SG dynamics, mitochondrial and endoplasmic reticulum homeostasis, and stress‐response pathways [2, 4, 77, 78]. Created in https://BioRender.com.

The physical and functional interactions between lncRNA‐encoded micropeptides and cytoplasmic SGs are most apparent during the integrated stress response (ISR) [4]. When eIF2α phosphorylation suppresses global cap‐dependent translation, alternative non‐canonical mechanisms, such as Internal Ribosome Entry Sites (IRES), upstream ORFs (uORFs), and N^6^‐methyladenosine (m^6^A) epitranscriptomic alterations, are selectively activated [6]. These specialised translation events commonly occur within or close to the shell of LLPS condensates, allowing for the fast synthesis of micropeptides exactly when and where they are needed for stress adaption [67, 76]. Once formed, these micropeptides function as micro‐switches, stabilising or destabilising RNP granules by influencing the molecular stoichiometry and phase separation thresholds of core nucleators, such as G3BP1/2 and TIA‐1 (Figure 2) [40, 77].

In malignant microenvironments, lncRNA‐encoded micropeptides play a critical role in cell fate determination, cell cycle regulation, and treatment resilience [19, 79]. These small proteins locate to essential sub‐cellular compartments, such as mitochondria, endoplasmic reticulum, and existing RNP complexes, to buffer stress signals under extreme conditions, including hypoxia, food deprivation, or chemotherapy [80]. For example, some stress‐induced micropeptides stabilise metabolic topologies and control the unfolded protein response in the mitochondria, which suppresses pro‐apoptotic cascades and increases resistance to chemotherapy [78]. On the other hand, by interacting with oncogenic pathways or removing proliferative factors from protective condensates, certain lncRNA‐derived peptides have been shown to behave as intrinsic tumour suppressors and halt cancer progression [47]. Together, these results highlight the importance of the micropeptide‐mediated layer of regulation for a thorough comprehension of cancer cell plasticity, providing new targets for targeted therapeutic intervention and novel diagnostic biomarkers.

#### 3.1.2 Long Non‐Coding RNAs as Stress Granule Modulators in Cancer Cells

LncRNAs affect SG dynamics via influencing the expression of SG‐associated proteins or by directly interacting with the SG axis [81]. For example, *MALAT1*, a well‐known lncRNA in cancer, regulates the localisation and dynamics of SG proteins such as G3BP1, which is essential for SG nucleation. By modulating SG stability, *MALAT1* enhances the survival of cancer cells under stress conditions [66, 82]. The dysregulation of membrane‐less organelle (MLO) formation affects chromatin organisation, oncogenic transcription, signalling pathways, and telomere length. Therefore, the lncRNA composition within MLOs dictates their activities and leads to dysregulation, contributing to human malignancies [3, 75].

Although many lncRNAs have been reported to modulate tumourigenesis, only the X‐inactive specific transcript (*XIST*) and *MALAT1* have been specifically linked to tumourigenesis. The direct association of these lncRNAs with SGs has not been studied in the context of cancer progression. However, it has been shown that *NEAT1* could be a potential target for promoting cancer cell sensitivity to chemotherapy in a p53‐dependent manner [83]. Metabolic rewiring during stress may also cause the activity of lncRNAs within the SGs to regulate survival mechanisms. For example, in the absence of glutamine, an increase in glutamine insufficiency regulator of glutaminase lncRNA (*GIRGL*) levels in the cell leads to the formation of a complex between glutaminase 1 (GLS1) mRNA and CAPRIN1. CAPRIN1 association promotes SGs and suppresses GLS1 mRNA translation, allowing cancer cells to survive [84, 85]. LncRNAs may compartmentalise SGs, thereby affecting gene expression linked to stress tolerance and cancer development. In addition, they interact with a variety of proteins and RNA molecules, thereby rewiring biological pathways and increasing cancer cell proliferation and survival under harsh conditions [79, 86, 87].

All of these findings support the growing idea that lncRNAs may actively influence the composition, stability, and function of SGs rather than being passive elements of stress‐responsive RNP assemblies. However, there are still a number of significant unanswered questions. Despite the fact that lncRNAs, such as *MALAT1*, *NEAT1*, and *GIRGL,* have been linked to stress adaptation and SG‐associated pathways, there is currently insufficient direct evidence indicating whether these transcripts serve as molecular scaffolds, structural SG constituents, or upstream regulatory factors. Moreover, it is yet unknown if unique lncRNA repertoires define functionally separate SG subtypes in cancer cells or whether SG‐associated lncRNAs display stress‐specific recruitment patterns. The discovery that metabolically responsive lncRNAs, like *GIRGL*, control SG formation further suggests that lncRNAs could act as molecular bridges between condensate biology and metabolic reprogramming. If these pathways are better understood, it may be possible to determine whether SG‐associated lncRNAs are downstream indicators of cellular stress responses or active drivers of cancer adaptability.

#### 3.1.3 Functional Implications of Long Non‐Coding RNAs in Stress Granule‐Driven Cancer Progression

LncRNAs influence the cellular capacity to adapt to stress by modulating both transcriptional and post‐transcriptional gene expression, which is crucial for cancer cells to endure and proliferate in harsh environments [80]. LncRNA‐mediated modulation of SGs has been associated with the development of cancer through several processes such as regulation of gene expression, modulation of therapy resistance, and remodelling of the tumour microenvironment [88]. By suppressing tumour suppressor genes or promoting oncogenic pathways, they enable cancer cells to evade apoptosis and proliferate [89]. Through their interactions with stress granule components such as G3BP, human antigen R (HuR), tristetraprolin (TTP), and ubiquitin‐specific peptidase 10 (USP10), lncRNAs regulate autophagy and chemoresistance by playing roles in different signalling pathways, from the tumour suppression p53 pathway to autophagy pathways like PI3K/AKT/mTOR. For example, cancer susceptibility candidate 2 (CASC2) inhibits autophagic processes, increasing sensitivity to chemotherapeutic treatments [90, 91, 92]. LncRNAs influence the tumour microenvironment by regulating metabolic processes and immunological responses, which are often associated with stress granule activity [88].

In summary, lncRNAs stand out as key regulators determining the adaptive capacity of tumour cells. By coordinating the dynamics of transcriptional and translational processes and ribonucleoprotein complexes, they modulate cellular senescence, metabolic remodelling, proliferative signalling, programmed cell death, and immune evasion. This regulatory network, integrating stress signals with post‐transcriptional mechanisms such as stress granule formation, shapes tumour progression and therapeutic response by adjusting the balance between drug resistance and cell death. This study focuses on apoptosis and drug resistance, two fundamental processes that determine the fate of cells under stress conditions. Apoptosis represents the failure of adaptive responses, while drug resistance refers to the maintenance of survival through the reprogramming of stress response pathways. In this context, we aim to address how lncRNA‐mediated stress granule dynamics shape survival networks through translational control components and how these networks are therapeutically modified and disrupted. LncRNAs that serve as modulators or drivers of the development of cancer are listed in Table 1 [19, 59, 80, 93, 94, 95, 96].

**TABLE 1:** The lncRNAs playing a role in cancer development.

lncRNAs	Cancer Types	Role in Cancer	Implications	Effect on SGs
*HULC*	Cholangiocarcinoma, Breast cancer	Spread of malignancy	Poor prognosis	Modulation in RNA‐binding protein (RBP) availability and stabilisation of oncogenic transcripts
*DLX6‐AS1*	Breast cancer	Tumour growth and metastasis	Tumour growth and metastasis	Reshaping SG composition
*TM4SF1‐AS1*	Gastric cancer	Preventing apoptosis	Tumour growth	Indirect effect on SG under stress conditions through modulating PI3K/AKT/mTOR signalling pathway
*H19*	Cholangiocarcinoma, Pituitary	Modifying translation and metabolic pathways	Reduced protein translation under metabolic stress	SG stabilisation through G3BP1 and HuR interaction
*GLCC1*	Colorectal cancer	Stabilisation of mRNAs under metabolic stress	Cancer cell survival	Stabilisation of c‐Myc mRNA within SG during metabolic stress
*SAMMSON*	Melanoma	Promoting mitochondrial activity under metabolic stress	Cancer cell survival	Altering ribonucleoprotein organisation, effect on proteostasis
*CCAT2*	Colon cancer	Remodelling of WNT/β‐catenin signalling	Tumour growth and spread of malignancy	Selective mRNA stabilisation within SGs
*BORG*	Breast cancer	Controlling cellular reactions under genotoxic stress	Cancer cell survival	Interaction with RBPs to enhance SG stability
*MALAT1*	Colorectal and non‐small cell lung cancer	Tumour growth and metastasis	Poor prognosis, tumour progression	Interaction with SG nucleating proteins to improve SG assembly and phase separation dynamics
*NEAT1*	Glioma, ovarian cancer, hepatocellular carcinoma, melanoma and prostate cancer	Tumourigenesis	Survival of oncogenic cells	Regulation in RBP granule organisation and phase separation processes

#### 3.1.4 lncRNAs In Stress Granules and Cell Cycle Regulation

Emerging evidence suggests that SG dynamics are closely linked to cell cycle regulation. Cellular stress frequently induces cell cycle arrest through activation of checkpoint pathways, allowing cells to conserve resources and repair damage. SGs contribute to this adaptive response by transiently sequestering untranslated mRNAs and regulatory proteins, thereby reshaping gene expression programmes during stress [32, 97]. Under conditions such as oxidative stress, hypoxia, nutrient deprivation, or chemotherapy, activation of the stress response leads to translational repression through eIF2α phosphorylation and promotes SG assembly. Simultaneously, stress activation modulates cell cycle checkpoints, particularly at the G1/S and G2/M transition. Activation of these checkpoints allows cells to halt proliferation and preserve cellular resources for damage repair and survival [4, 19].

Several SG‐associated RBPs are involved in cell cycle regulation. For example, G3BP1, as a central SG nucleator, influences the stability and translation of transcripts implicated in the proliferation and survival pathways. Increased G3BP1 expression has been linked to increased tumour growth and cell cycle progression in a variety of cancer types. Similarly, TIA1 and CAPRIN1 promote selective mRNA sequestration during stress, influencing the availability of transcripts encoding cyclins, cyclin‐dependent kinases (CDKs), and checkpoint regulators. Through these processes, SGs can temporarily block the translation of mRNAs that promote cell cycle progression while encouraging cellular stress adaptability [39].

LncRNAs appear to serve as key intermediaries between SG dynamics and cell cycle control. *MALAT1* regulates the expression of many cell cycle‐associated genes, including B‐MYB and other transcriptional regulators essential for G1/S progression and mitotic entry. *MALAT1* depletion causes cell cycle arrest and reduced proliferation, emphasising its involvement in coordinating cell cycle progression. Similarly, *MALAT1*‐mediated modulation of RBPs and stress‐responsive pathways suggests that it may have an effect on SG‐associated post‐transcriptional regulation [98]. *NEAT1* is transcriptionally activated by p53 in response to cellular stress. *NEAT1* promotes paraspeckle formation and helps with replication stress adaptation, DNA damage response, and chemosensitivity. Because SGs and paraspeckles communicate functionally during stress, *NEAT1* could act as a molecular bridge between stress‐induced RNA compartmentalisation and cell cycle checkpoint activation. Indeed, stress‐induced SG formation has been demonstrated to enhance paraspeckle assembly by upregulating *NEAT1*, which contributes to a coordinated stress adaptation network [83]. *H19* modulates MYC, E2F, and Wnt/β‐catenin signalling pathways to enhance proliferation and cancer stem cell maintenance, affecting cell cycle progression. Similarly, *TM4SF1‐AS1*, which increases SG assembly by binding G3BP2 and RACK1, reduces apoptosis and improves stress tolerance in cancer cells. Although its direct effect on cell cycle checkpoints is unknown, its potential to extend SG persistence may indirectly aid survival during stress‐induced proliferative arrest [66, 95].

These data support a concept in which lncRNAs use stress sensing, SG dynamics, and cell cycle regulation to improve cancer cell survival. By coordinating checkpoint activation, translational repression, and adaptive gene expression programmes, SG‐associated lncRNAs may assist tumour cells in balancing brief growth arrest with long‐term proliferative capacity. Future research combining single‐cell transcriptomics, RNA interactome mapping, and live‐cell imaging is required to determine the precise molecular mechanisms that support this emergent regulatory network.

### 3.2 Long Non‐Coding RNAs in Suppression of Stress Granule‐Mediated Apoptosis in Cancer Development

The precise significance of the SG function in cancer cells remains unclear due to its confounding effect under different conditions, namely, acting as both an oncogenic and a tumour suppressor [50]. The assembly of SGs under stress conditions protects osteosarcoma cells from apoptosis by modulating intrinsic apoptotic pathways, activating caspase 3/7, and cleaving poly (ADP‐ribose) polymerase (PARP) [99]. SGs' ability to prevent cellular senescence and apoptosis by sequestering PAI‐1 and pro‐apoptotic proteins, such as TRAF2 or RACK1, may help cancer cells survive, while the downregulation of USP10, which is an SG component, has tumour‐suppressive function in hepatocellular carcinoma [50]. It was shown that caspase‐3/7 accumulates in SGs through evolutionarily conserved amino acid residues within their extensive catalytic domains. Consequently, this accumulation inhibits caspase activity and the apoptosis induced by diverse stressors, such as ER stress and UV induction [99].

The recruitment of apoptotic factors into stress granules is not merely a passive confinement process; rather, these granules act as dynamic regulatory nodes that temporally regulate cell fate. The selective recruitment of pro‐ and anti‐apoptotic proteins is driven by multivalent protein–protein/RNA interactions regulated mainly by RACK1, TIA‐1, some mTOR signalling components, and TRAF‐2; post‐translational regulation, and RNA‐mediated structural organisation mainly by *NEAT‐1* and *NORAD* [95, 99].

Non‐canonical SGs, such as those generated under UV, may be pro‐apoptotic, whereas canonical SGs often perform a cytoprotective function by circumventing apoptosis [16]. Accordingly, SGs may house pro‐apoptotic mRNAs and proteins, such as Early Growth Response 1 (EGR1) mRNA, Receptor For Activated C Kinase 1 (RACK1), p38, and JNK pathway proteins, thereby preventing cancer cells from undergoing apoptosis [91]. Certain lncRNAs strengthen the apoptotic effect by directly blocking apoptosis‐related pathways in SGs. SGs promote the storage of essential mRNAs in cancer cells, inhibit stress‐induced apoptosis, and recruit regulatory RNAs, such as lncRNAs [39]. For example, *HOTAIR* has been shown to increase survival under oxidative stress, allowing cancer cells to evade apoptosis [100, 101]. LncRNAs *H19* and *HULC*, acting as microRNA sponges and being up‐regulated in cholangiocarcinoma cells exposed to oxidative stress, enable cancer cells to survive against oxidative stress by increasing the release of cytokines that facilitate invasion and migration [80]. The nuclear hypoxia‐regulated *NLUCAT1* lncRNA enables cells to resist ROS‐induced apoptosis by promoting the expression of oxidative homeostasis genes such as *ALDH3A1*, *GPX2*, *GLRX*, and *PDK4* in lung adenocarcinoma cells. Additionally, depletion of *NLUCAT1* renders cells more susceptible to oxidative stress‐induced cell death [80]. Forkhead Box D3 Antisense RNA‐1 (*FOXD3‐AS1*), an oncogene associated with oxidative stress in gliomas, is thought to regulate oxidative stress pathways to promote survival [102].

The fascinating concept that lncRNAs may contribute to the functional specialisation of different SG populations is supported by the growing distinction between canonical cytoprotective SGs and non‐canonical pro‐apoptotic SGs. To ascertain whether particular lncRNA signatures characterise SGs that support survival, apoptosis, or therapeutic resistance, future research combining SG purification, spatial transcriptomics, single‐molecule imaging, and functional perturbation techniques will be crucial. By addressing these issues, it may be possible to gain a better understanding of how SG composition affects the fate of cancer cells and if lncRNA‐mediated manipulation of SG dynamics can be targeted therapeutically. Whether tumour‐suppressive lncRNAs aid in the creation of non‐canonical pro‐apoptotic condensates while oncogenic lncRNAs are preferentially enriched in cytoprotective SGs is a significant unanswered question. Tackling this option might uncover a novel and functional selectivity layer in SG biology.

### 3.3 Long Non‐Coding RNAs in Stress Granule Formation and Therapy Resistance

LncRNAs modulate the formation, content, and activity of SGs, impacting cancer cell survival and drug resistance [47, 103]. Many malignancies survive chemotherapy and radiation, owing in part to SGs' protective effect. For example, lncRNA *LINP1* has been linked to therapeutic resistance by stabilising SGs, lowering drug‐induced oxidative stress, and increasing DNA repair [104]. LncRNAs may influence cell survival during therapeutic stress by modulating the degradation of pro‐apoptotic mRNAs within SGs [80, 105]. The PKR/eIF2α pathway is a crucial regulator of SG formation. Phosphorylated eIF2α leads to the formation of cytoplasmic aggregates through polysome disassembly and accumulation of RNA‐binding proteins and untranslated mRNAs, which protects cancer cells against the stress‐induced damage [106, 107, 108].

**FIGURE 3:**
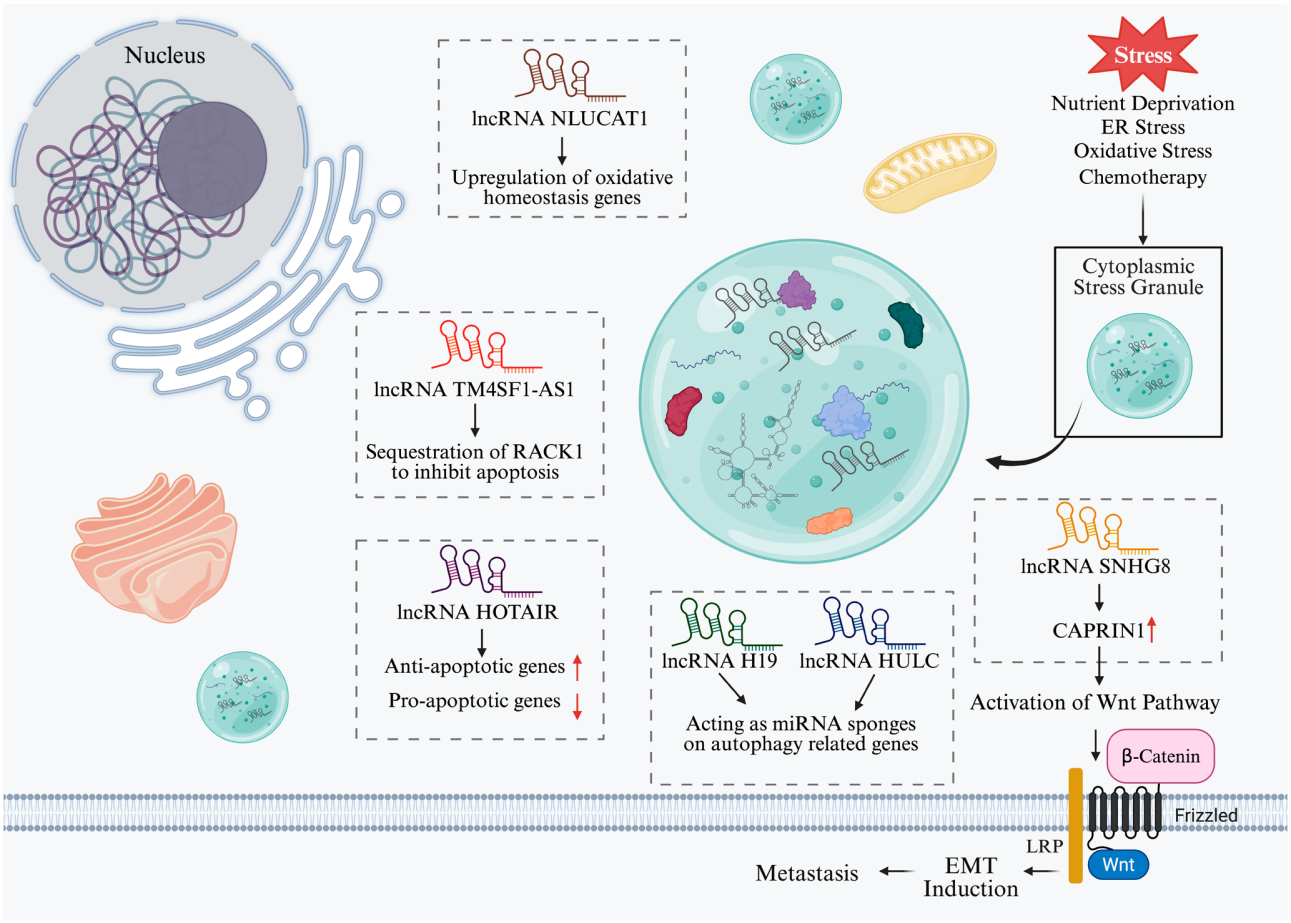
LncRNAs modulate tumour progression through different mechanisms. HOTAIR and TM4SF1‐AS1 suppress apoptotic processes by binding to the protein components of stress granules and thereby increasing the survival capacity of tumour cells [100, 101]. LncRNAs such as NLUCAT1 play a critical role in the modulation of oxidative stress responses by affecting the redox homeostasis of cancer cells. Their interaction with autophagy pathways allows cancer cells to develop adaptive resistance to chemotherapy [80]. In addition, lncRNAs can modulate EMT induction through increasing expression of EMT transcription factors by activated Wnt/β‐Catenin pathway regulating EMT‐related gene expression [114, 115]. Created in https://BioRender.com.

Invasion, self‐renewal, and survival are among the key characteristics of cancer stem cells (CSCs), and these features are enhanced by lncRNAs modulating the stemness‐associated transcriptional factors such as SOX2, Nanog, and Oct‐4 and signalling pathways such as Notch, Hedgehog, and Wnt/β‐catenin [109, 110, 111]. *MALAT1* upregulates anti‐apoptotic proteins (e.g., IAPs, Bcl‐2 family) and drug resistance in glioblastoma. Similarly, lncRNA *NEAT1* promotes chemoresistance and CSC populations in triple‐negative breast cancer [112]. Epithelial‐Mesenchymal Transition (EMT), a process in which epithelial cells acquire mesenchymal and migratory characteristics, is closely associated with the development of CSC and resistance to chemotherapy [110, 113]. LncRNA *linc‐ROR* is known to promote drug resistance and EMT processes, which promotes survival and metastasis through activated EMT transcription factors or *linc‐ROR*‐sponged miRNAs. Several mechanisms of EMT progression through lncRNAs are presented in Figure 3 [116, 117].

Some lncRNAs allow CSCs to evade chemotherapy or radiotherapy by regulating the expression of DNA repair enzymes and drug‐efflux transporters such as MRP1 and ABCB5. For example, lncRNA *AFAP1‐AS1* stimulates Notch signalling, increasing cisplatin resistance [111, 113]. Under hypoxic or nutrient‐poor tumour microenvironments, lncRNAs may promote CSC survival and energy requirements by regulating glycolysis and other metabolic pathways. LncRNA *H19* enhances glycolysis by upregulating *PDK1*, thereby enhancing the stemness characteristics and chemoresistance of breast cancer [112]. In addition, as a key regulator of cancer stem cell development and maintenance, lncRNA *H19* contributes to and regulates the tumour‐initiating capacity, metastatic and self‐renewal potential of CSCs via different mechanisms, including miRNA sponging, activating oncogenic pathways, and modulating stemness genes [114, 115].

All of these findings point to a diverse set of molecular mechanisms by which lncRNAs contribute to treatment resistance that go beyond the control of specific signalling pathways. LncRNAs seem to play a key role in coordinating stress tolerance in cancer cells by affecting stemness programmes, SG dynamics, metabolic adaptability, and DNA damage responses. However, a number of significant questions are still unresolved. The degree to which chemoresistance and radioresistance are directly mediated by SG‐dependent pathways is still mostly unknown, despite the fact that many lncRNAs have been linked to these traits. Specifically, it is unclear if SGs mainly function as downstream effectors of lncRNA‐driven stemness programmes or if SG‐associated lncRNAs actively enhance the acquisition of cancer stem cell features. Furthermore, it is still unclear what molecular factors control the selective recruitment of regulatory RNAs and resistance‐associated transcripts into SGs under therapeutic stress. SG‐associated lncRNAs may serve as integrative hubs, integrating hypoxia tolerance, metabolic reprogramming, and cancer stem cell maintenance, according to emerging findings.

#### 3.3.1 Methodological Strategies for Examining the Relationships Between lncRNAs and Stress Granules

Combining imaging, biochemical, transcriptomic, and proteomic methods is necessary to fully comprehend the role of lncRNAs in SG biology. A single approach is insufficient to completely characterise lncRNA–SG interactions due to the dynamic and varied nature of SGs. To identify SG‐associated lncRNAs, to ascertain their spatial localisation, to identify their interacting partners, and to explore their functional contributions to stress‐induced biomolecular condensates, complementary approaches are typically used.

Immunofluorescence (IF) microscopy remains the most frequently used method for detecting and quantifying SGs. SGs are often visualised with antibodies to conventional SG markers, such as G3BP1, G3BP2, TIA‐1, TIAR, eIF3, and PABP. Quantitative image analysis allows for the evaluation of SG number, size, cellular distribution, and assembly/disassembly dynamics under various stress situations. Advances in live‐cell imaging with fluorescently tagged SG proteins have allowed for real‐time visualisation of condensate nucleation, fusion, maturation, and dissolution dynamics in living cells. These methods are especially relevant for studying how lncRNA depletion or overexpression impacts SG formation and persistence [118, 119].

RNA fluorescence in situ hybridisation (RNA‐FISH) allows for direct visualisation of individual lncRNAs at single‐cell resolution. When paired with immunofluorescence labelling for SG markers, RNA‐FISH serves as a useful tool to find out if lncRNAs of interest are spatially localised within SGs. This method has been widely employed to study the subcellular localisation of stress‐responsive transcripts and the stress‐induced redistribution of RNAs between the nucleus and the cytoplasm. Single‐molecule FISH (smFISH) is considerably more sensitive and enables quantitative investigation of transcript abundance and SG recruitment at the individual RNA level [62]. Because SGs include a high number of RBPs, characterising lncRNA‐RBP interactions is crucial for understanding their biology. RNA immunoprecipitation (RIP) is a typical method for detecting RNAs linked with SG proteins including G3BP1, TIA‐1, FUS, and TDP‐43. Advanced approaches, including crosslinking and immunoprecipitation (CLIP), PAR‐CLIP, iCLIP, and eCLIP, allow for high‐resolution mapping of RNA‐protein interaction sites across the transcriptome. These techniques provide crucial information on the sequence motifs and structural variables that govern lncRNA recruitment into SG‐associated RNP complexes [120, 121].

Recent advances in proximity labelling have significantly improved the ability to characterise the composition of SGs. APEX‐seq, APEX‐MS, BioID, TurboID, and CAP‐seq are methods for identifying RNAs and proteins that are in the vicinity of SG components in living cells. These methods can capture transitory and low‐affinity interactions that are sometimes difficult to detect with conventional biochemical techniques. Proximity labelling has emerged as a powerful method for finding hitherto unrecognised SG‐associated lncRNAs and studying the molecular architecture of stress‐induced condensates [122, 123]. Super‐resolution imaging techniques, such as STORM, PALM, SIM, and lattice light‐sheet microscopy, have shown that SGs have intricate interior architectures rather than representing homogeneous structures.

These methods enable the visualisation of SG subcompartments, nanoscale architecture, and RNA‐protein distributions. Fluorescence recovery after photobleaching (FRAP) is a common method for assessing molecular mobility inside SGs and determining how individual lncRNAs affect condensate material characteristics, liquidity, and exchange dynamics. Such approaches are particularly useful for studying LLPS mechanisms underpinning SG assembly [61, 72]. Transcriptomic and proteomic profiling techniques are increasingly used to characterise SG‐associated RNAs and proteins in depth. Using SG purification, RNA sequencing (RNA‐seq), mass spectrometry, ribosome profiling, and single‐cell transcriptomics, thousands of SG‐enriched transcripts and proteins have been identified. Such investigations have demonstrated that transcript length, translational state, RNA structure, and RBP‐binding capacity all influence SG recruitment. These high‐throughput techniques are especially useful for discovering novel lncRNAs linked with SGs and understanding how SG composition changes across different stress situations and cancer types [62, 124].

Future research combining high‐resolution imaging, proximity labelling, single‐cell transcriptomics, and spatial multi‐omics technologies is expected to provide novel insights into how lncRNAs regulate SG assembly, composition, and function in cancer and other stress‐related diseases. Relatively few lncRNAs have been functionally verified as genuine regulators of SG assembly or activity, despite the fact that transcriptome and proteomic investigations have discovered a large number of SG‐associated RNAs and proteins. Moreover, several widely utilised methods, such as assays based on RIP and CLIP, mainly identify molecular proximity or binding events rather than establishing causal roles in condensate formation. These drawbacks emphasise the need for integrated experimental frameworks that incorporate functional perturbation techniques, such as single‐cell analysis, high‐resolution live‐cell imaging, and spatial transcriptomics.

Another important question that begs for further research is whether diverse lncRNA profiles may be used to identify functionally unique stress granule populations. Approaches that combine biophysical characterisation with multi‐omics datasets may be useful in addressing if certain lncRNAs selectively control pro‐apoptotic, therapy‐resistant, or cytoprotective SG states. Answering this question could greatly improve our knowledge of SG heterogeneity and make it easier to identify clinically relevant lncRNA–SG networks that could be used as therapeutic targets or diagnostic biomarkers.

### 3.4 Therapeutic Potential of Targeting Long Non‐Coding RNA‐Mediated Stress Granule Formation

Targeting SG‐associated lncRNAs represents a promising therapeutic approach, given SGs' pro‐survival role in cancer. As shown in Table 2, targeting lncRNAs involved in SG dynamics may help disrupt SG formation, reduce cancer cell resistance, and enhance the efficacy of current cancer treatments [13, 39, 66, 83, 93, 105, 125, 126]. CRISPR‐mediated editing, antisense oligonucleotides, and RNA interference (RNAi) are all novel therapeutic approaches to target and silence lncRNA‐driven SG formation mechanisms. Nucleic acid‐based methods, such as antisense oligonucleotides (ASOs) or small interfering RNAs (siRNAs), can inhibit SG‐mediated survival by targeting oncogenic lncRNAs (e.g., *TM4SF1‐* *AS1* and *NEAT1*) [95]. For example, ASOs have been designed to alter the *NEAT1* isoform balance (by boosting tumour‐suppressive *NEAT1_2*) [127]. A recent study identified drugs (G3Ia/G3Ib) that bind to the G3BP1/2 pocket and prevent SG formation. By blocking SG nucleators, these chemicals break down existing SGs and make cells more susceptible to stress. Small compounds could also potentially target lncRNA structures or RBP interactions; however, such drugs are currently experimental [77, 127].

**TABLE 2:** The role of SG‐related lncRNAs as a therapeutic implication.

lncRNA	Cancer association(s)	SG‐related function	Therapeutic implication
*TM4SF1‐AS1*	Gastric cancer	Binds Pur‐α and YB‐1, recruits SG proteins (G3BP2, RACK1), sequesters RACK1 into SGs to inhibit apoptosis	ASO/siRNA knockdown may restore RACK1 signalling and apoptosis
*GIRGL*	Colorectal cancer	Under glutamine starvation, scaffolds CAPRIN1 dimers and GLS1 mRNA, inducing CAPRIN1 phase separation and SGs, suppressing GLS1 translation	Targeting GIRGL could sensitise cells to metabolic stress or inhibit SG‐mediated survival
*P53RRA (LINC00472)*	Lung, breast (p53‐wildtype)	Binds G3BP1 (SG protein), displacing p53 from G3BP1 complexes, increasing nuclear p53 and inducing apoptosis/ferroptosis	Mimicking P53RRA or G3BP1 inhibitors could activate the p53 pathway in tumours
*PHAROH (Gm19705)*	Hepatocellular carcinoma	Sequesters the SG/RBP TIAR via a 71‐nt hairpin, relieving TIAR‐mediated translational repression of MYC, thereby elevating MYC protein	ASO against PHAROH may reduce MYC‐driven proliferation^4^
*NEAT1*	Multiple (glioma, ovarian cancer, hepatocellular carcinoma, melanoma and prostate cancer)	Core scaffold of nuclear paraspeckles; upregulated by stress signals including those from SGs. Enhances stress‐adaptive gene regulation	Targeting NEAT1 isoforms (e.g., ASOs) could disrupt stress‐responsive gene networks
*NORAD*	Various; involved in genomic stability	Recruited into SGs during viral infection, where it is cleaved by RNase L. May connect DNA damage signalling with cytoplasmic SGs	Modulating NORAD levels may alter p53/interferon signalling and stress responses
*SNHG8*	Ovarian carcinoma (and others)	Binds and upregulates CAPRIN1 expression (an SG nucleator), promoting Wnt signalling and potentially SG‐related survival pathways	Inhibiting SNHG8 could disrupt CAPRIN1‐driven SG functions and downstream oncogenic signalling

Other approaches include altering lncRNA loci using CRISPR, modulating upstream stress kinases (e.g., eIF2α inhibitors) to indirectly influence SGs and mimicking RNAs or using aptamers to sequester RBPs. In conclusion, both nucleic acid therapeutics (ASOs/siRNAs) and new small‐molecule condensate modulators offer promising avenues for regulating lncRNA‐mediated SG dynamics [93, 127, 128]. In a preclinical study, Lipid Nanoparticle (LNP)‐encapsulated siRNAs against oncogenic lncRNAs such as *LINC01257* hold promise for the treatment of paediatric acute myeloid leukaemia (AML). While specific SG/lncRNA‐targeted drugs are not yet in trials, advances in lncRNA delivery (ASO/siRNA) and SG‐modulating small molecules have paved the way for future interventions. Once the link among lncRNAs, SGs, and cancer has been unequivocally documented, we anticipate that targeted therapeutics (e.g., ASOs against SG‐promoting lncRNAs or condensate inhibitors) will progress toward clinical evaluation [127].

Targeting the lncRNA–SG axis for therapy is gaining popularity, but before these approaches can be translated into clinical settings, several significant issues need to be resolved. First, there are issues with specificity and possible off‐target consequences because many SG‐associated lncRNAs participate in several cellular pathways outside of SG regulation and display context‐dependent roles. Second, there is yet little direct mechanistic data showing that therapeutic suppression of these lncRNAs impairs SG‐dependent stress tolerance, even though several lncRNAs have been connected to SG formation and cancer cell survival. The existence of functionally different SG subtypes with pro‐apoptotic or cytoprotective characteristics raises another unanswered question: are all SG populations equally acceptable as therapeutic targets? Therefore, unexpected biological outcomes could result from indiscriminate disruption of SGs. To identify the most clinically relevant lncRNA–SG networks and differentiate actionable treatment targets from secondary stress‐response markers, future research combining functional genomics, spatial transcriptomics, and condensate‐specific proteome investigations will be essential. The lncRNA–SG axis may become a unique class of precision medicine targets that can overcome medication resistance and improve treatment outcomes across various cancer types as RNA‐based therapies and condensate‐targeting methods continue to progress.

#### 3.4.1 Emerging Treatment Strategies for the lncRNA‐Stress Granule Axis

SGs have drawn a lot of attention as prospective therapeutic targets due to their increasing recognition as essential regulators of cellular adaptability. Interventions targeted at impairing SG formation or function may be viable cancer treatment approaches because of their proven involvement in tumour survival, therapeutic resistance, metabolic adaptation, and stress tolerance. Additionally, a growing body of research indicates that lncRNAs have a role in SG composition, assembly, and signalling, underscoring the lncRNA–SG axis as a potentially druggable regulatory network. Strategies that target SG dynamics, lncRNA‐mediated pathways, RNA–protein interactions, and LLPS mechanisms can be used to broadly classify emerging treatment approaches.

Direct suppression of SG assembly has become a popular therapeutic approach since many cancer cells depend on SG production to withstand chemotherapy, hypoxia, and oxidative stress. Pharmacological disruption of SG nucleators, such as G3BP1 and TIA‐1, has been shown to impair stress tolerance and increase the vulnerability of cancer cells to cytotoxic therapy. Furthermore, substances that alter translational control mechanisms, ISR pathways, or eIF2α signalling may indirectly prevent SG development and improve therapy efficacy. The therapeutic value of SG‐targeting strategies has been supported by that inhibition of SG assembly can sensitise tumour cells to chemotherapy and decrease adaptive stress responses [129, 130, 131, 132].

Targeting disease‐associated lncRNAs has become more feasible with the advent of RNA‐based treatments. Oncogenic lncRNAs linked to cancer growth and treatment resistance have been selectively suppressed by ASOs, siRNAs, short hairpin RNAs (shRNAs), CRISPR/Cas‐based technologies, and RNA‐targeting CRISPR systems. Because of their roles in stress adaptation, DNA damage responses, and RNA regulatory networks, several SG‐associated or stress‐responsive lncRNAs, such as *NEAT1*, *MALAT1*, *NORAD*, and *LINP1*, have been suggested as possible therapeutic targets. Specifically, ASO‐mediated *MALAT1* suppression has demonstrated an encouraging anticancer effect in preclinical animals, indicating the viability of therapeutically targeting lncRNAs [133, 134].

Interactions between lncRNAs and RBPs are essential for many biological processes. As a result, lncRNA–RBP complex disruption has become a potentially effective treatment approach [135]. The synthesis of tiny compounds that can disrupt particular RNA–protein interactions has been made easier by developments in structural biology and RNA‐targeted drug discovery [136]. Pharmacological disruption of these interaction networks may destabilise SGs and hinder stress adaptation because SG formation is mostly dependent on multivalent interactions between lncRNAs and SG‐associated RBPs, such as G3BP1, FUS, TIA‐1, TDP‐43, and hnRNP proteins. Despite the fact that the majority of these strategies are still in preclinical phases, they offer a new therapeutic intervention option [74, 137, 138, 139, 140, 141].

A new treatment avenue has been made possible by the discovery that SGs are assembled via LLPS. Modulation of condensate material qualities may change SG formation and persistence because LLPS relies on weak multivalent interactions involving IDRs, RNA molecules, and RNA‐binding proteins. Weak hydrophobic interactions that stabilise biomolecular condensates are disrupted by small compounds, such as 1,6‐hexanediol. Thus, it is possible to pharmacologically disrupt phase‐separated structures, even though their toxicity and nonspecific effects challenge their use in therapeutic settings. More recently, efforts have focused on developing selective modulators of condensate formation, condensate viscosity, and protein phase behaviour. Eventually, these approaches might make it possible to specifically interrupt diseased SGs while maintaining normal cellular condensates [72, 76]. Considering the complexity of SG biology, combined therapies will probably be one of the most successful approaches. Targeting oncogenic lncRNAs and SG assembly pathways simultaneously may increase the tumour's susceptibility to immunotherapy, chemotherapy, and radiation. SG‐directed therapeutics may also work in concert with immune checkpoint inhibitors and other immunotherapeutic methods, as new research indicates that SGs play a role in immune evasion and adaptation within the tumour microenvironment.

Selective alteration of lncRNA–RBP interactions may represent a novel strategy to impede condensate formation and overcome stress‐adaptive phenotypes in cancer cells [74, 140, 141]. There is increasing evidence that stress granules are diverse structures whose biological roles and composition might change based on the cell type, the stress stimuli, and the illness environment. Because SGs play physiological roles in preserving cellular homeostasis, broad suppression of SG assembly may not always be beneficial. Selectively delivering RNA‐based treatments to tumour tissues while reducing off‐target effects in healthy cells is a significant additional obstacle.

## 4 Conclusions

**FIGURE 4:**
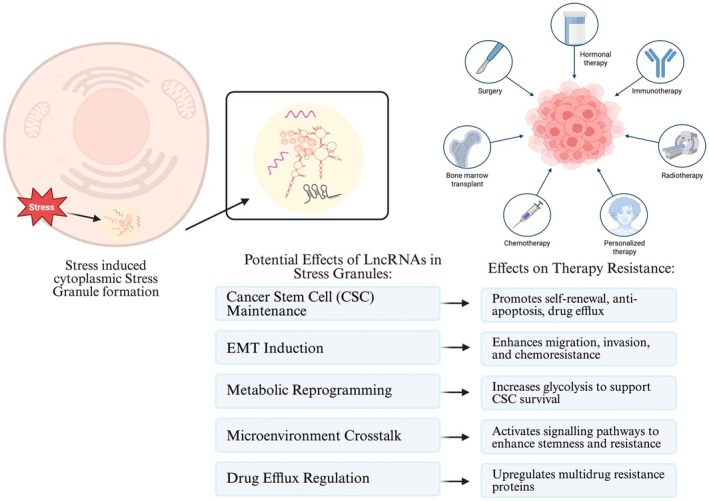
Integrated model for lncRNAs in SGs in the manner of their potential effects and therapy resistance mechanisms. Under cellular stress conditions, cytoplasmic stress granules (SGs) are formed, incorporating lncRNAs that influence various cancer‐related processes. These include cancer stem cell (CSC) maintenance, epithelial‐to‐mesenchymal transition (EMT), metabolic reprogramming, microenvironmental crosstalk, and drug efflux regulation. Through these mechanisms, lncRNAs in SGs potentially promote self‐renewal, enhance invasiveness, increase glycolytic activity, activate pro‐survival signalling, and upregulate multidrug resistance, ultimately contributing to resistance against diverse cancer therapies. Created in https://BioRender.com.

LncRNAs play critical roles in regulating SG formation and dynamics, thereby influencing the survival, adaptation, and resistance of cancer cells to therapy. By modulating SG assembly and stabilising key RBPs, such as G3BP1/2, TIA‐1, FUS, TDP‐43, and Fragile X Messenger Ribonucleoprotein 1 (FMRP), within these structures, lncRNAs enhance cancer cells' resilience to environmental stressors. LncRNAs enable tumours to thrive in harsh environments by interacting with these RBPs, regulating oxidative and genotoxic stress responses, and stabilising pro‐survival genes. Understanding these processes holds promise for overcoming therapeutic resistance by providing new targets for the lncRNA‐SG axis [88, 125, 142]. To translate these discoveries into targeted treatments, future studies must provide functional validation of potential lncRNAs and prioritise the development of RNA‐targeted therapies. These multifaceted roles are illustrated in Figure 4, which summarises how lncRNAs within stress granules contribute to the maintenance of cancer stem cells, metabolic reprogramming, and therapeutic resistance mechanisms. Understanding these processes offers new avenues for overcoming therapeutic resistance by targeting the lncRNA–SG axis [143].

SGs in cancer cells exhibit significant differences, both qualitatively and quantitatively, from those observed in healthy cells. In particular, the types of lncRNAs, oncogenic factors, and pro‐survival mRNAs display a unique SG composition in cancer cells. This diverse composition not only supports survival under cellular stress conditions but also mediates maintenance of cell proliferation, suppression of apoptosis, and strengthening of therapy resistance mechanisms [19, 38]. In line with the changing cellular stress environment, the composition and functional heterogeneity of SGs change as cancer progresses. SG components may differ between early and late‐stage tumours, reflecting the tumour cells' requirements for survival, proliferation, invasion, and resistance to therapy. These modulations promote cancer cell survival, proliferation, metastasis, and resistance mechanisms [19, 78]. Differential expression across tumour stages for SG‐specific lncRNAs, such as *circ‐CREIT*, *SNHG8*, and *TM4SF1‐AS1* is observed in several studies [19, 65, 95]. While late‐stage tumours exhibit upregulation of lncRNAs that influence SG assembly to enhance survival, invasion, and therapy resistance, early‐stage tumours may exhibit little SG‐associated lncRNA activity. However, the distinct lncRNA composition in core and shell compartments of SGs across tumour stages, and how these distinct characteristics may contribute to cancer progression have remained unclear.

Although the structural architecture of SGs appears to be conserved between normal and malignant cells, current findings reveal that SGs in cancer cells undergo a compositional and biophysical remodelling. Abnormal expression of canonical nucleators such as G3BP1 and TIA1 in tumours lowers the phase separation threshold, increasing the persistence of stressed condensations and triggering apoptosis resistance [7, 40, 78]. Specifically, cytoplasmic HuR (ELAVL1) accumulation provides selective stabilisation of survival‐related transcripts within SGs, directing the post‐transcriptional regulatory network toward oncogenic outcomes [144].

In accordance with the principles of biomolecular condensation biology, protein concentration, multivalency, and RNA abundance are critical parameters determining the component properties of phase‐separated structures [76]. In this context, oncogenic lncRNAs, such as *MALAT1*, *NEAT1*, *H19*, and *HULC* function as structural scaffolds that reshape SG dynamics. For example, *NEAT1* facilitates condensation formation through highly valuable RNA‐protein interaction domains under stress conditions, while *MALAT1* modulates translational control mechanisms through the redistribution of RBPs [145, 146].

Consequently, the increasing stoichiometry of core proteins and oncogenic lncRNAs shifts SGs from dynamic and reversible fluid‐like structures to more persistent and rigid condensations. This type of pathological stabilisation chronicises the suppression of pro‐apoptotic mRNAs while optimising the preservation of survival and metabolism‐focused transcripts, thereby strengthening therapeutic resistance. Current data support the hypothesis that cancer‐associated SGs represent context‐dependent maladaptive condensations that promote tumour progression rather than simple physiological adaptation mechanisms [76, 147].

Classification of SG‐associated oncogenic lncRNAs according to their genomic biotypes reveals the predominance of intergenic scaffold‐type transcripts (lincRNAs) with functional links to stress response RBP networks as seen in Table 3 [9, 14, 86, 105, 108]. This classification shows that, in addition to lincRNAs, antisense and “sense‐overlapping” lncRNAs also actively participate in SG modulation; however, the roles of enhancer and intronic transcripts in this context remain largely undefined [55, 76, 145, 148].

**TABLE 3:** Potential effects of lncRNA biotypes on stress granule formation.

lncRNA biotypes	Mechanisms	SG‐related interaction
Antisense	Cis‐regulation, mRNA stability	RBP competition, mRNA preservation
lincRNA	Scaffold, trans‐regulation	Phase separation, RNP organisation
Enhancer RNA	Transcriptional activation	Indirect ISR activation
Intronic lncRNA	Host gene related	Local RBP modulation
Sense‐overlapping	mRNA processing	Translational control

The specific molecular contributions of lncRNAs to the assembly, composition, and functional heterogeneity of SGs in cancer are still unclear, despite rising evidence that lncRNAs are involved in SG biology. In our view, future studies should move beyond descriptive associations and focus on identifying direct molecular interactions between lncRNAs and SG nucleators, such as G3BP1/2, TIA1, CAPRIN1, and DDX3X. Development of high‐resolution approaches, such as single cell transcriptomics, spatial transcriptomics, RNA interactome capture, and live‐cell imaging, will likely uncover novel lncRNA‐dependent regulatory networks within SGs. Furthermore, the selective targeting of oncogenic SG‐associated lncRNAs using ASOs, siRNAs, CRISPR‐based technologies, or small molecules that disrupt RNA‐protein condensates may provide new opportunities to overcome therapy resistance. However, significant challenges remain, including tumour‐specific delivery, off‐target effects, and the dynamic nature of SG composition. We anticipate that the integration of biomolecular condensate biology with RNA therapeutics will establish SG‐associated lncRNAs as a new generation of potential targets in cancer treatment and precision oncology.

## 5 Outstanding Questions

Whether particular functional groups of lncRNAs show preferential enrichment within SGs is an interesting but mainly unanswered subject in SG biology. Interestingly, a number of lncRNAs that are now linked to SG dynamics, such as *NEAT1*, *NORAD*, *MALAT1*, and *LINP1*, have been linked to genome stability, tumour growth, stress adaptation, or treatment resistance. These findings suggest that lncRNAs may be selectively recruited into stress granules under cellular stress, oncogenic or pro‐survival conditions. Such selective enrichment may empower cancer cells with a quick and reversible tool to maintain vital networks of RNA–protein interactions, coordinate adaptive gene expression programmes, and improve survival in harsh microenvironments [61, 62]. Nevertheless, the existing data are insufficient to unequivocally state whether SGs prefer oncogenic lncRNAs over tumour‐suppressive lncRNAs. In fact, very few transcriptomic studies have examined the lncRNA makeup of SGs in various cellular settings. As a result, it is unclear if tumour‐suppressive lncRNAs are simply under‐represented in SGs or if they contribute to SGs biology through different, as yet poorly understood processes [32, 53].

Selective lncRNA recruitment into SGs may be influenced by a number of molecular variables. These include the ability of individual lncRNAs to engage in multivalent RNA–protein interaction networks, transcript quantity, RNA length, sequence composition, secondary structure, and interaction affinity with stress granule‐nucleating RBPs, such as G3BP1, TIA‐1, and FUS. Additionally, the composition of SGs is very dynamic and varies depending on the cell type, the stress stimuli, and the disease state. This suggests that the enrichment of lncRNAs is context‐dependent [64, 74]. To ascertain whether functional selectivity exists among SG‐associated lncRNAs, future research utilising proximity‐labelling techniques, single‐cell transcriptome analysis, and SG‐specific RNA sequencing will be highly crucial.

## Author Contributions

**Nazlı Şevval Menemenli:** writing – review and editing, writing – original draft, conceptualization, visualization. **Bünyamin Akgül:** conceptualization, supervision, funding acquisition, writing – review and editing.

## Funding

This study is funded by the Scientific and Technological Research Council of Turkey (TUBITAK Project No: 113Z371 to BA).

## Conflicts of Interest

The authors declare no conflicts of interest.

## Data Availability

Data sharing not applicable to this article as no datasets were generated or analysed during the current study.
